# Multi-Sensor Fusion for Underwater Vehicle Localization by Augmentation of RBF Neural Network and Error-State Kalman Filter

**DOI:** 10.3390/s21041149

**Published:** 2021-02-06

**Authors:** Nabil Shaukat, Ahmed Ali, Muhammad Javed Iqbal, Muhammad Moinuddin, Pablo Otero

**Affiliations:** 1 Oceanic Engineering Research Institute, University of Malaga, 29010 Malaga, Spain; ahmedali@uma.es (A.A.); mjavediqbal99@uma.es (M.J.I.); pablo.otero@uma.es (P.O.); 2 Department of Electrical and Computer Engineering, King Abdulaziz University, Jeddah 21589, Saudi Arabia; mmsansari@kau.edu.sa; 3 Center of Excellence in Intelligent Engineering Systems, King Abdulaziz University, Jeddah 21589, Saudi Arabia

**Keywords:** underwater vehicle, navigation, multi-sensor fusion, localization, RBF, underwater robotics

## Abstract

The Kalman filter variants extended Kalman filter (EKF) and error-state Kalman filter (ESKF) are widely used in underwater multi-sensor fusion applications for localization and navigation. Since these filters are designed by employing first-order Taylor series approximation in the error covariance matrix, they result in a decrease in estimation accuracy under high nonlinearity. In order to address this problem, we proposed a novel multi-sensor fusion algorithm for underwater vehicle localization that improves state estimation by augmentation of the radial basis function (RBF) neural network with ESKF. In the proposed algorithm, the RBF neural network is utilized to compensate the lack of ESKF performance by improving the innovation error term. The weights and centers of the RBF neural network are designed by minimizing the estimation mean square error (MSE) using the steepest descent optimization approach. To test the performance, the proposed RBF-augmented ESKF multi-sensor fusion was compared with the conventional ESKF under three different realistic scenarios using Monte Carlo simulations. We found that our proposed method provides better navigation and localization results despite high nonlinearity, modeling uncertainty, and external disturbances.

## 1. Introduction

The ocean floor has billions of dollars of natural resources in the form of precious elements and medicinal herbs. To take advantage of ocean resources, seabed mapping is the ultimate tool that depends on precise sensors and robust navigation fusion algorithms for autonomous underwater vehicles (AUVs) and remotely operated underwater vehicles (ROVs). Navigational accuracy is a key requirement for complex seabed mapping tasks [1]. However, primary sensors, three-axis gyros, and accelerometers have biases and drifts, which vary with time and are affected by noise. In contrast, most commonly used fusion algorithms based on extended Kalman filter (EKF) and its variant error-state Kalman (ESKF) suffer from divergence and degraded mean square error (MSE) performance in the nonlinear underwater condition because of linear approximation [2]. Thus, the higher the nonlinearity present in the system, the greater the error of the EKF state prediction, and it can also induce filter divergence.

Backpropagation multi-layer neural networks (BPNN) and radial basis neural networks (RBFNN) have excellent learning abilities and are well-known for their nonlinear system identification [3,4,5]. In comparison, the RBFNN has much greater accuracy of prediction and versatility in their choice of base functions [6]. Furthermore, they have fast convergence and less computation load compared to BPNN. These advantages of RBFNN lead to a major research question: Can we incorporate the strengths of the RBFNN to improve the underwater vehicle localization performance of ESKF?

### 1.1. State-of-the-Art Review

The basic form of an on-board navigation system on any underwater vehicle comprises an inertial measurement unit (IMU) that can determine positions by integrating three-axis acceleration and angular velocities [7,8]. This basic form of navigation suffers from drift, typically 1.8 km per day to 1.5 km per hour based on the grade of IMU [7,9,10], which makes them practically impossible to use for long missions. On top of that, most common off-board positioning by global positing system (GPS) satellites does not work underwater because of radio frequency attenuation [11]. Alternate communication means based on acoustic positioning are widely used underwater, which suffers from communication uncertainly and delays [12,13]. On-board aiding sensors, Doppler velocity log, pressure sensor, and magnetometers can also help to reduce the effect of IMU drift, but all these sensors are affected by noise. To improve the navigation accuracy and to minimize disturbance because of noise, EKF-based algorithms are the most commonly used in underwater navigation and localization [14,15,16,17,18,19].

On the other hand, the EKF algorithm has its shortcoming in that the accuracy of estimation is reduced under high nonlinear system dynamics. However, many variants of EKF have been proposed in academic research to cater to this problem [20,21]. Most of the EKF and neural network estimation algorithms are designed for land-based vehicles. In these approaches, when GPS data is present and valid, the neural network is trained and, when GPS information is not available, the neural network output improves the EKF prediction. For instance, a detailed study [22] proposed a hybrid offline trained RBFNN with time series prediction for measurement update during GPS outage. Another study [23] combined extreme learning machine neural network (ELM) and EKF to bridge the GPS outage. They claimed to have a better real-time performance by improving the computation load. In addition, some authors [24,25] also suggested using machine learning techniques to improve localization via intelligent communication networks, but their research is limited to land-based applications. Recently, an intelligent methodology was proposed that uses deep learning neural networks with EKF [26]. Moreover, they claimed that, by using recurrent neural networks (RNNs), state estimation can be improved and their model can also work well with low-cost sensors. Nevertheless, they did not incorporate oceanic parameters for state prediction besides the fact that RNNs have a high computational cost.

Another researcher group, in [27], used underwater model-aided dead reckoning to improve EKF response. They calculated aided velocity using an identified surge dynamic model. This work design reached a position accuracy of 92% during external position fix outage. However, using a model in this design makes the system difficult to tune under different sensors and working conditions because models depend on various factors such as size, the weight of the AUV, and the physical characteristics of the sensors. The authors of [23] proposed to embed underwater vehicle dynamic equations in the EKF and estimated error in the navigation. This work claimed to have less computation load and better accuracy compared to a full model integration. However, it is also dependent on the physical parameters of the vehicle and has more implementation complexity.

Likewise, a study [28] compared underwater EKF and its statistical linearization variant, also known as unscented Kalman filter (UKF) [29] in their project. They found that the statistical form provides better accuracy in highly nonlinear conditions. Similar results were found in [30]. Nonetheless, the UKF drawbacks include implementation complexity, high computational time and cost, and round-off error [31,32].

Traditionally, the Kalman filter is used to train RBFNN [33,34] or in offline training of the radial basis function (RBF) [35]. These methods underperform in uncertain conditions with unmodeled dynamics. The authors of [36] introduced a forgetting factor, which is based on RFBNN, to improve the performance of central difference Kalman filter (CDKF) for attitude-of-the-satellite estimation. They proposed the range of forgetting at 0.2 to 2 as a multiplier to the Kalman gain, but limited description of the selection of the forgetting factor was provided. Recently, the RBFNN-aided Kalman Filter was proposed to improve the state estimation accuracy for spacecraft navigation [37]. Moreover, they did not use multi-sensor fusion of high-rate and low-rate sensors.

### 1.2. Contributions of the Paper

The proposed work fills the gap by proposing a novel multi-sensor fusion architecture based on the strengths of the RBF neural network and error-state Kalman filter for underwater navigation, which has not been proposed to date to the authors’ knowledge. We named this algorithm RBF-ESKF. The augmentation of both algorithms improves the navigation of underwater vehicles in GPS-less environments. Our major contribution is the derivation of a multi-sensor fusion algorithm that improves the accuracy of underwater localization by taking advantage of a radial basis function (RBF) neural network that has the capability of nonlinear universal approximation via recursive learning [38]. Moreover, a simple structure of the RBF network can be trained online with less computation cost compared to the backpropagation neural network (BPNN) [39].

The structure of this paper is organized as follows. Section 2 discusses kinematic mathematical modeling with an ellipsoid Earth model. Section 3 discusses external and internal sensors for underwater navigation. In addition, the mathematical models of the sensors are described with their operating noise characteristics. The error dynamic model is presented in Section 4. In Section 5, a multi-sensor fusion algorithm is derived for accurate underwater integrated navigation. Section 6 shows the test results of the proposed multi-sensor fusion filter in three different conditions. In addition, acoustic communication lost and on-board sensor malfunctioning are tested and analyzed. Moreover, the performance of the RBF-ESKF is compared with ESKF under different scenarios. A comparative analysis is performed, showing that the proposed algorithm has promising results.

## 2. Mathematical Modeling

This section discusses the mathematical modeling of the underwater vehicle taken from [40,41,42,43,44]. The first subsection explains the notations used for modeling. The second subsection describes the frame of references used for the mathematical formulation of IMU. The third subsection formulates the kinematics equation of motion for six degrees of freedom. The last subsection provides brief information on the sensors used for underwater navigation.

### 2.1. Frame of References

**Figure 1:**
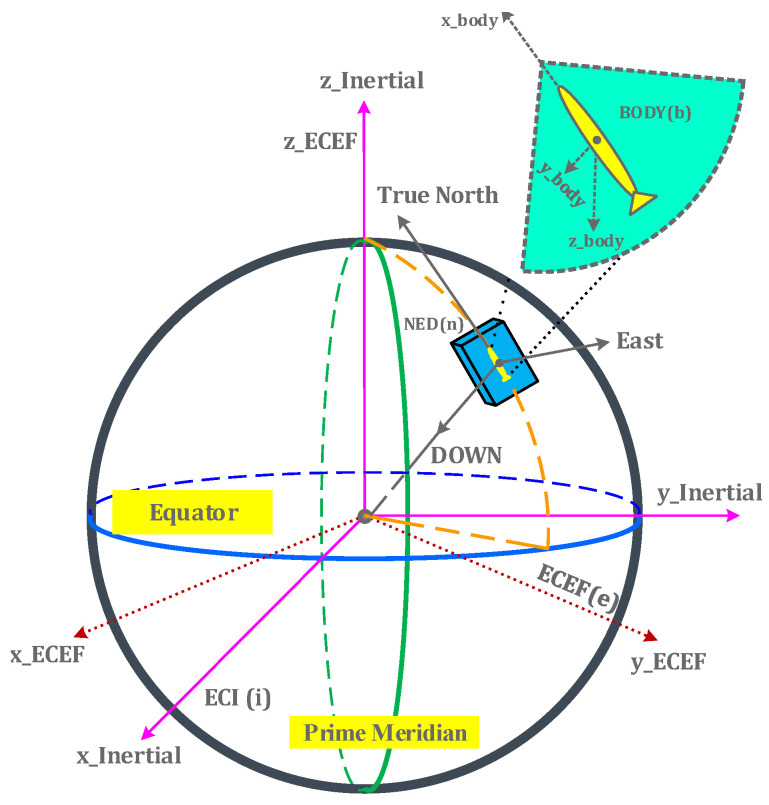
Frames of references: ECI, ECEF, NED and Body.

The importance of frame of reference transformation for underwater navigation arises from the fact that sensors are mounted on the vehicle’s body. The origin of the body is defined as the center of body frame (b) External position fixes are in a rotating, Earth-centered, Earth-fixed (ECEF) frame (e). Moreover, Newton’s laws are applicable to the Earth-centered inertial (ECI) frame (i). However, the north-east-down (NED) frame or navigation frame (n) is the tangent plane to the Earth’s surface at the location of the underwater vehicle and its x-axis points toward the true north of the Earth. Figure 1 shows a frame of references used to develop navigation equations for underwater vehicles, with slight modification from [40].

### 2.2. Mathematical Notation

The mathematical notations used in this work are standard notations used to model underwater vehicle position, velocity, and attitude [40]. The position and velocity are three dimensional (3D) vectors in Euclidean space. Rotations are represented by quaternion. Subscripts and superscripts are used to represent the relationship between the frames. For example, $\omega_{ie}^{e}$ shows an angular velocity of frame (e) with respect to (i) represented in (e) frame. The following Table 1 shows a list of symbols used in the development of underwater vehicle navigation equations.

### 2.3. Navigation Equations

The underwater vehicles are most commonly equipped with a strapdown inertial navigation system [41]. In this configuration, measurements are obtained directly in the body frame and sensors experience full rotation during maneuvers. Moreover, this configuration requires an initial condition for position, velocity, and attitude. The kinematics equations for the underwater vehicles used in this work are described in [42,43,44]. The rate of change in position $\dot{p^{e}}$ and velocity $v^{e}$ of vehicle in (e) frame is related by the following differential equation:

(1) $${\dot{p}}^{e}=v^{e}$$

The rate of change in velocity $\dot{v^{e}}$ of the underwater vehicle in (e) frame is dependent on accelerometer output $f^{b}$, angular velocity $\omega_{ie}^{e}$, and gravity vector $g^{e}$ in ECEF and can be expressed by the following differential equation:

(2) $$\dot{v^{e}}=R_{b}^{e}f^{b}-2\Omega_{ie}^{e}v^{e}+g^{e}$$

where $\Omega_{ie}^{e}$ is a skew symmetric matrix of angular velocity $\omega_{ie}^{e}$ and $R_{b}^{e}$ is the rotation matrix that transforms a specific force vector from the (b) frame to the (e) frame.

The rate of change of rotation matrix ${\dot{R}}_{b}^{e}$ represented in frame (e) is dependent on angular velocity $\Omega_{ib}^{b}$ of the body with respect to frame (i), and angular velocity $\omega_{ie}^{b}$ of Earth with respect to frame (i) is expressed by the following equation:

(3) $${\dot{R}}_{b}^{e}=R_{b}^{e}\left(\Omega_{ib}^{b}-\Omega_{ie}^{b}\right)$$

The relationship between the change in latitude of the vehicle $\dot{\phi_{lat}}$ and velocity is represented by the following differential equation:

(4) $$\dot{\phi_{lat}}=v_{N}/R_{M}+h_{d}$$

where $R_{M}=\frac{a}{\sqrt{1-e^{2}\sin\phi }}$

The change in longitude of the vehicle ${\dot{\lambda }}_{long}$ in the form of the east velocity is represented by following mathematical relationship:

(5) $${\dot{\lambda }}_{long}=v_{E}/\left(R_{N}+h_{d}\right)\cos\phi_{lat}$$

where $R_{N}=R_{M}\frac{1-e^{2}}{\sqrt{1-e^{2}\sin\phi_{lat}}}$

The change in height of the vehicle $\dot{h_{d}}$ is expressed in the form of the down velocity as

(6) $$\dot{h_{d}}=-v_{D}$$

The latitude $\phi_{lat}$, longitude $\lambda_{long}$, and depth $h_{d}$ are given as

(7) $$\phi_{lat}=\tan^{-1}\left[\frac{z/\sqrt{x^{2}+y^{2}}}{1-e^{2}R_{M}/\left(R_{M}+h_{d}\right)}\right]$$

(8) $$\lambda_{long}=\tan^{-1}\left[y/x\right]$$

(9) $$h_{d}=\frac{\sqrt{x^{2}+y^{2}}}{\cos\phi_{lat}}-R_{M}$$

The rate of change in velocity of the vehicle in the (n) frame ${\dot{v}}_{eb}^{n}$ is expressed by the following differential equation:

(10) $${\dot{v}}_{eb}^{n}=R_{b}^{n}f_{in}^{b}-\left(\Omega_{en}^{n}+2\Omega_{ie}^{n}\right)v_{eb}^{n}+g_{eb}^{n}$$

where $\Omega_{en}^{n}v^{n}$ is the centripetal acceleration related to the motion of the (n) frame with respect to the (e) frame and $2\Omega_{ie}^{n}v^{n}$ is the Coriolis acceleration.

The local gravity vector $g_{eb}^{n}={\left[\begin{array}{ccc} 0 & 0 & g \end{array}\right]}^{T}$ depending on the latitude, longitude, radius of curvature of the meridian, and radius of curvature of the prime vertical is given by

(11) $$g_{eb}^{n}=\frac{g_{0}}{{\left(1+\frac{h_{d}}{\sqrt{R_{N}R_{M}}}\right)}^{2}}$$

where $g_{0}=9.780318\times \left(1+5.3024\times 10^{-3}\sin^{2}\phi -5.9\times 10^{-6}\sin^{2}2\phi \right)$ [44]. The rate of change in the rotation matrix ${\dot{R}}_{b}^{n}$ represented in frame (n) is dependent on the angular velocity of the body with respect to frame (i) and on the angular velocity of frame (n) with respect to frame (i):

(12) $${\dot{R}}_{b}^{n}=R_{b}^{n}\left(\Omega_{ib}^{b}-\Omega_{in}^{b}\right)$$

The attitude of the vehicle is represented by quaternion. It has only one constraint compared to the direction cosine matrix (DCM), and this method is also singularity free in comparison to the Euler angle representation, which has a singularity problem [45].

The attitude of the vehicle represented in quaternion ${\dot{q}}_{b}^{n}$ is given as

(13) $${\dot{q}}_{b}^{n}=\frac{1}{2}q_{b}^{n}⊗\left[\begin{array}{c} o\\ \omega_{ib}^{b} \end{array}\right]-\frac{1}{2}\left[\begin{array}{c} 0\\ \omega_{in}^{n} \end{array}\right]⊗q_{nb}$$

where $q_{b}^{n}$ has two parts: η is the scalar part; $\epsilon_{i}$ is the vector part; and *i* = 1,2,3. The ⊗ sign represents a quaternion product.

This section developed the motion equations of an underwater vehicle in starpdown configuration. The measurements from accelerometers were obtained in (b) frame as a specific force vector and were not the true acceleration of the vehicle. To obtain the true acceleration or rate of change in velocity of the vehicle in *e* frame, as shown by Equation (2), the (b) frame specific forces are transformed into the *e* frame by a rotational matrix and by compensating for the gravitation and rotation effects of the Earth. Equation (1) shows that the rate of change in the position and acceleration has an integral relationship. Since the position from an acoustic fix is obtain in the geoid ECEF frame, for compatibility, Equations (4)–(9) convert the position of the vehicle in latitude, longitude, and depth. Equation (13) shows a quaternion representation of the roll, pitch, and yaw angles of the vehicle. Interested readers can find more details for mathematical modeling of an underwater vehicle in starpdown configuration in [40,41,42,43,44].

## 3. Sensors on the Vehicle

The following subsections give a brief overview of the sensors used in the AUV with a mathematical formulation of errors. For detail about the mathematical model of the sensors used for navigation, readers can refer to [40,46,47].

### 3.1. Inertial Measurement Unit

The inertial measurement unit (IMU) provides a three-axis accelerometer and three-axis gyro outputs [48]. These measurements are affected by variable factors such as temperature, manufacturing process, scale factor, noise, and drift.

The accelerometer measurement output vector $f_{acc}^{b}$ in (b) frame is modeled as

(14) $$f_{acc}^{b}=f_{ib}^{b}+b_{acc}+\varrho_{acc}$$

where $\varrho_{acc}$ is white noise and $b_{acc}$ is acceleration bias, which is modeled as a 1st-order Markov process.

The accelerometer bias $b_{acc}$ is expressed by the random walk and the random constant shown as

(15) $${\dot{b}}_{acc}=-\tau_{acc}^{-1}b_{acc}+\rho_{acc}$$

where $\tau_{acc}$ is the correlation time given by the manufacturer. Its value depends on the accelerometer sensors used in the IMU. The value $\rho_{acc}$ depends on standard deviation, and it is known as a process driving noise.

The accelerometer in IMU does not directly measure the true kinematic acceleration of vehicles due to the presence of Earth’s gravitation. For that reason, the measurement from the accelerometer is known as the relative acceleration or specific force $f_{ib}^{b}$ and it is related to the kinematics acceleration of the vehicle as follows:

(16) $$f_{ib}^{b}={\dot{R}}_{i}^{b}{\ddot{p}}^{i}-g^{b}$$

where ${\ddot{p}}^{i}$ is acceleration represented in an inertial frame and $g^{b}$ is gravitation sensed by the accelerometer in the body frame.

The actual output of the gyro $\omega_{g}^{b}$ is influenced by noise and bias that is given by

(17) $$\omega_{g}^{b}=\omega_{ib}^{b}+b_{g}+\varrho_{g}$$

where $\varrho_{g}$ is the white noise and $b_{g}$ is the gyro bias, which is modeled as a 1st-order Markov process.

The gyro bias $b_{g}$ is represented by a random walk and a random constant given as follows:

(18) $${\dot{b}}_{g}=-\tau_{g}^{-1}b_{g}+\rho_{g}$$

where $\tau_{g}$ is the gyro correlation time obtained from manufacturer documentation. Its value depends on the quality of the accelerometer sensors used in the IMU. The value $\rho_{g}$ depends on thte standard deviation, and it is called process driving noise. The IMU used for simulation is tactical grade Emcore SDI-1500. It uses high-precision micro-electro-mechanical systems (MEMS) quartz sensor technology with 1${}^{\circ }$/h gyro bias and 1 mg accelerometer bias stability. The IMU offers the best cost to performance ratio compared to other technologies. It consists of three orthogonal accelerometer sensors that provide the measurement of specific forces. Three gyros provide the angular rates of the body with respect to the inertial frame of reference.

### 3.2. Underwater Acoustic Positing System

The underwater acoustic positing system measures the distance and direction of the vehicle from the reference positions. For this work, HiPaP 502 is used for simulation. This system provides a typical range detection accuracy of 0.2 m, with an operating range of 1 to 5000 m. It has an acoustic operating area of 200${}^{\circ }$/200${}^{\circ }$ with the capability of narrow beamforming of 10${}^{\circ }$, which improves the signal-to-noise ratio. It can be interfaced by GPS to provide Earth-related coordinates. However, acoustic position estimate is effected by GPS accuracy, system installation, ship attitude, sound velocity profile, ray bending, and measurement noise.

Assuming the system is precisely calibrated, installation and ship attitude have negligible effects. The mathematical model of the system actual output $\hat{p}$ is given as

(19) $${\hat{p}}_{h}=p_{h}+b_{h}+\varrho_{h}$$

where $p_{h}$ is true output position, whereas $b_{h}$ is the time-varying bias modeled as a 1st-order Markov process and depends on the sound velocity profile and ray bending effect. $\varrho_{h}$ represents measurement white noise.

### 3.3. Doppler Velocity Log

The Doppler velocity log (DVL) measures the change in acoustic frequency for determining the speed of the vehicle with reference to the seabed. In deep water, when the seabed is not available, the DVL measures the speed with respect to water. The DVL sends a known frequency signal to the seabed and receives the signal that bounces back to the vehicle. The speed of the underwater vehicle is a dependent doppler effect [49]. The work used Nortek DVL-500, which has a range of 0.3 to 200 m and long-term accuracy of ±0.1% ±0.1 cm/s. The DVL can be operated in a 4-beam Janus configuration complete performance testing of DVL; readers may refer to [50].

Considering that the attitude and installation error are negligible, the actual output of DVL ${\hat{v}}_{dvl}$ can be modeled for the true velocity measurement vector, noise, and bias given as [51].

(20) $${\hat{v}}_{dvl}=v_{dvl}+b_{dvl}+\varrho_{dvl}$$

where $v_{dvl}$ is the true output and random velocity error, $b_{dvl}$ is modeled as the 1st-order Markov process, and $\varrho_{dvl}$ is white noise.

### 3.4. Depth Sensor

Depth and underwater pressure have a direct relationship [52]. As the vehicle goes deep into water, the pressure reading increases linearly. The depth sensor modeled in this work is from Paroscientific, Inc. part number 8CDP700-I, which provides an accuracy of 0.01% and high stability under tough conditions.

The depth sensor actual output ${\hat{h}}_{d}$ is modeled by adding true depth $h_{d}$ with noise:

(21) $${\hat{h}}_{d}=h_{d}+\varrho_{d}$$

where $\varrho_{d}$ is measurement noises modeled as white noise.

### 3.5. Magnetometer

The magnetometer or compass measures the magnitude and direction of Earth’s magnetic field [53]. The magnetometer used in the work is jewel instrument ECS-AC-RS232 e-compass, which has a 3-axis magnetometer and a 2-axis tilt sensor. The tilt sensor is used for the initialization of roll and pitch [40]. It offers an accuracy of ±0.5${}^{\circ }$ root mean square RMS, a repeatability of ±0.3${}^{\circ }$, and a response time of 36 milliseconds. The pitch and roll are ±42${}^{\circ }$ whereas the dip angle range ±80${}^{\circ }$. The major error sources of the magnetometer include the declination angle, which is the difference between true north and sensor north; the hard and soft magnetic distortion due to motors and ferromagnetic materials [54]; and sensor imperfection, misalignment, and noise. However, with proper calibration, most of the errors in measurement can be removed.

The actual output $q_{m}$ of the magnetometer is a combination of noise and true output $q_{m}$ written as

(22) $${\hat{q}}_{m}=q_{m}⊗\varrho_{m}$$

where $\varrho_{m}$ is the sensor noise modeled as white noise.

## 4. Error Dynamic Model

The position p, velocity v, attitude q, gyro bias $b_{g}$, accelerometer bias $b_{acc}$, and acoustic fix bias $b_{h}$ in full state vector x form are given as

(23) $$x={\left[\begin{array}{ccccccc} p & v & q & b_{g} & b_{acc} & b_{h} & s_{v} \end{array}\right]}^{T}$$

where $s_{v}$ is the sound velocity model underwater used to calculation underwater acoustic transmission.

The estimated state vector $\hat{x}$ is written as

(24) $$\hat{x}={\left[\begin{array}{ccccccc} \hat{p} & \hat{v} & \hat{q} & {\hat{b}}_{g} & {\hat{b}}_{acc} & {\hat{b}}_{h} & \hat{s_{v}} \end{array}\right]}^{T}$$

The design used in this worked estimate an error-state vector, which offers the main advantages of flexible sampling rate, robustness, and low computation burden [55,56]. The error-state vector $\delta \dot{x}$ is the difference between the true state and estimated state $\dot{\hat{x}}$ of the model and is given as

(25) $$\delta x=x-\hat{x}$$

The error-state vector can be represented as

(26) $$\delta x={\left[\begin{array}{ccccccc} \delta p & \delta v & \delta q & \delta b_{g} & \delta b_{acc} & \delta b_{h} & \delta s_{v} \end{array}\right]}^{T}$$

where the position, velocity, and attitude error equations are provided as under. For complete derivation of this error model, readers can refer to [42,57].

Assuming that the underwater vehicle travel at low speed and that the depth of operation is much less than the Earth’s radius, the rate of change in errors in longitude $\delta \dot{\lambda_{long}},$ latitude $\delta \dot{\phi_{lat}}$, and depth $\delta \dot{h_{d}}$ are given by

(27) $$\begin{array}{c} \left[\begin{array}{c} \delta {\dot{\lambda }}_{long}\\ \delta {\dot{\phi }}_{lat}\\ \delta \dot{h_{d}} \end{array}\right]=\left[\begin{array}{ccc} 0 & 0 & \frac{-v_{n}}{{\left(R_{N}+h_{d}\right)}^{2}}\\ \frac{v_{e}\sin\left(\lambda_{long}\right)}{\left(R_{N}+h_{d}\right)\cos^{2}\left(\lambda_{long}\right)} & 0 & \frac{-v_{e}}{{\left(R_{N}+h_{d}\right)}^{2}\cos\left(\lambda_{long}\right)}\\ 0 & 0 & 0 \end{array}\right]\left[\begin{array}{c} \delta \lambda_{long}\\ \delta \phi_{lat}\\ \delta h_{d} \end{array}\right]\\ +\left[\begin{array}{ccc} \frac{1}{R_{M}+h_{d}} & 0 & 0\\ 0 & \frac{1}{\left(R_{N}+h\right)\cos\left(\lambda_{long}\right)} & 0\\ 0 & 0 & -1 \end{array}\right]\left[\begin{array}{c} \delta v_{n}\\ \delta v_{e}\\ \delta v_{d} \end{array}\right] \end{array}$$

The velocity error $\delta {\dot{v}}_{eb}^{n}$ is given by the following differential equation:

(28) $$\begin{array}{cc} \delta {\dot{v}}_{eb}^{n}= & \delta g_{eb}^{n}+\delta R_{b}^{n}f_{acc}^{b}+R_{b}^{n}\delta f_{ib}^{b}-\left(2\left(\delta \Omega_{ie}^{n}\right)+\left(\delta \Omega_{en}^{n}\right)\right){\hat{v}}_{eb}^{n}-\left(2\left(\Omega_{ie}^{n}\right)+\left(\Omega_{en}^{n}\right)\right)\delta v_{eb}^{n} \end{array}$$

If the vehicle operates at low speed underwater, angler velocity $\omega_{ie}^{n}$ and $\delta \omega_{ie}^{n}$ can be neglected. Gravity error is also neglected because of the small operating area and accurate estimation [44,58].

Thus, the velocity error $\delta {\dot{v}}_{eb}^{n}$ differential equation can be re- written as

(29) $$\begin{array}{c} \delta {\dot{v}}_{eb}^{n}=\delta R_{b}^{n}f_{acc}^{b}+R_{b}^{n}\delta f_{ib}^{b} \end{array}$$

Assuming that the angular velocity of rotation of Earth with respect to the inertial frame $\omega_{ie}$ is accurately known, using the attitude error model in the form of the quaternion, it is given as

(30) $$\dot{\delta q}=\frac{1}{2}\delta \omega_{ib}^{b}+\frac{1}{2}\left(\delta \Omega_{ib}^{b}\right)\delta q$$

The error of gyro bias $\delta {\dot{b}}_{g}$ is given as

(31) $$\delta {\dot{b}}_{g}={\dot{b}}_{g}-{\dot{\hat{b}}}_{g}$$

The error of accelerometer bias $\delta b_{acc}$ is given as

(32) $$\delta b_{acc}={\dot{b}}_{acc}-{\dot{\hat{b}}}_{acc}$$

The error of hydro-acoustic system bias $\delta b_{acc}$ is given as

(33) $$\delta b_{h}={\dot{b}}_{h}-{\dot{\hat{b}}}_{h}$$

Since the ESKF employed in this work used an error-state or indirect-form-state vector z, it is obtained by subtracting the outputs of the inertial measurement unit (INS) and aiding sensors measurement [59].

The position error $\delta z_{p}$ between the INS position $p_{INS}$ and acoustic position system measurement $p_{h}$ is written as

(34) $$\delta z_{p}=p_{INS}-p_{h}$$

The velocity error $\delta z_{v}$ between the INS velocity $v_{INS}$ and DVL measurement $v_{d}$ is given by the following equation:

(35) $$\delta z_{v}=v_{INS}-v_{d}$$

The attitude error $\delta z_{v}$ between the INS attitude $q_{INS}$ and magnetometer measurement $q_{m}$ is not a vector quantity; it cannot be subtracted as position and velocity. Quaternion multiplication ⊗ is used to find the error term as follows:

(36) $$\delta z_{q}=q_{m}^{-1}⊗q_{INS}$$

## 5. RBF-ESKF Mulit-Sensor Fusion

**Figure 2:**
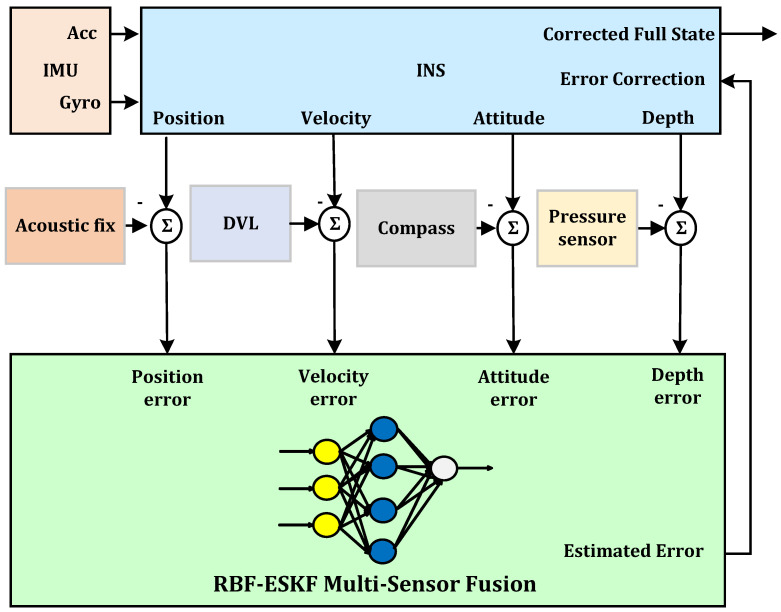
Top level diagram of the RBF-ESKF multi-sensor fusion navigation architecture.

The proposed modifications improve ESKF performance and make use of the advantages of the RBF neural network. The RBF neural network can approximate any nonlinear function, and they are also known as universal function approximators [60]. The RBF center, its width, and the linear weights for each output neuron are altered at every iteration of a learning algorithm. When each RBF center is as close to the input vector as possible and the network output error is within the target limit, the training phase is completed. Therefore, it is possible to express the approximation of any functional dependency between variables as a linear combination of a best possible number of RBF neurons with appropriate weight and center. The top level block diagram of our proposed fusion algorithm is depicted in Figure 2.

As shown by Figure 2, the algorithm takes the error of the aiding sensors and INS as input. The RBF-ESKF fusion algorithm after processing gives an output to INS for error correction and reset. The RBF neural network used for processing has a basic three layer structure; input, hidden, and output layer. Furthermore, compared to BPNN, the RBF variants have less computational load and fast online learning. The first layer is the input layer, which provides an interface between the data and neural network. The data from the input layer to the second hidden layer is transferred in such a manner that the output value of every hidden neuron is inversely related to the Euclidean distance from that neuron’s input vector to the RBF neuron’s center. The third layer is the output layer, which takes into account the cumulative weights and biases of all RBF neuron outputs.

Several variants of RBF exist in literature that depend on the application [61]. However, in this work, we used the Gaussian-type RBF function. The weights of neurons in the hidden layer for the Gaussian RBF function indicate the center of the symmetrical Gaussian distribution curve. The novelty of the RBF-ESKF algorithm is that it includes system information within the weight and center learning update rules.

### 5.1. RBF-ESKF Mathematical Formulation

The underwater vehicle navigation system has nonlinear dynamics and measurement characteristics, which can be represented in state space form by Equation (37) and Equation (38) [40]

(37) $$\dot{x}\left(t\right)=f\left(x\left(t\right),u\left(t\right),t\right)+\alpha \left(t\right)$$

where the state of the system is represented by $\dot{x}\left(t\right)$. The u(t) is a known system input and α(t) is Gaussian white noise.

The measurement output z(t) in state space can be written as

(38) z(t)=h(x(t),t)+β(t)

where f and h are nonlinear functions. The measurement is corrupted by Gaussian white noise β(t).

Dropping time and noise in the above equations for simplification, the linear form of error state can represented by Equation (39) and Equation (40): [40]

(39) $$\delta \dot{x}=F\left(\hat{x},u,t\right)\delta x$$

where $F\left(\hat{x},u,t\right)={\left.\frac{\partial f}{\partial x}\right|}_{x=\hat{x}}$

However because the deterministic component $F(\hat{x},u,t)$, is always incomplete, which means the model does not incorporate, for instance, AUV underwater movements due to waves, the stochastic component α(t) takes these effects into account.

The linear residual measurement output δz can represented by

(40) $$\delta z=H(\hat{x},t)\delta x$$

where $H\left(\hat{x},t\right)={\left.\frac{\partial h}{\partial x}\right|}_{x=\hat{x}}$, $\hat{x}$ is defined as the trajectory obtained by using vehicle kinematics equations.

The Kalman filter [62], in its basic form, is based on linear system and measurement models, which in reality might not be the case. In our underwater vehicle, navigation equations are used to construct the mathematical model and are not linear with respect to the state variables. This is done by linearization of any predicted trajectory, leading to an error-state model as discussed in Section 4. This linear approximation was proven to be incomplete and requires special consideration in underwater navigation, which leads to derivation of the RBF-ESKF algorithm.

For mathematical formulation of RBF-ESKF, we start with ESKF implementation, which utilizes definitions of measurement error and state dynamics error, as described by the Equations (39) and (40) [63]. The ESKF algorithm has three major components. The first component is initialization, in which state, covariance, process, and measurement covariance are initialized [64].

$x_{0}^{-}=$ Initialization of the state variables.
$P_{0}^{-}=$ Initialization covariance matrix.
$Q_{0}=$ Initialization process noise covariance.
$R_{0}=$ Initialization of measurement noise covariance.

where superscript minus ${}^{-}$ denotes the a priori state that occurs before innovation is updated. Superscript plus ${}^{+}$ denotes the posteriori state after innovation calculation.

The second component is time update, in which the error state and error-state covariance are updated, give by

(41) $$\delta x_{k+1}^{-}=\Phi_{k}\delta x_{k}^{-}$$

where $\delta x_{k+1}^{-}$ is the predicted error state and $\Phi_{k}$ is the state transition matrix in discrete form.

(42) $$P_{k+1}^{-}=\Phi_{k}P_{k}^{+}\Phi_{k}^{⊤}+Q_{k}$$

where $P_{k+1}^{-}$ is the predicted error covariance and $Q_{k}$ is the process noise covariance $v_{k}$.

The third component is measurement update, in which the residual of measurement is updated. The residual of measurement $\delta z_{k}$ is given by the difference between the actual measurement $z_{k}$ and the prediction of measurement $h\left({\hat{x}}_{k}\right)$

(43) $$\delta z_{k}=z_{k}-h\left({\hat{x}}_{k}\right)$$

The kalman $K_{k}$ gain is given as

(44) $$K_{k}=p_{k}^{-}H_{k}^{⊤}{\left(H_{k}p_{k}^{-}H_{k}^{⊤}+R_{k}\right)}^{-1}$$

The posteriori error estimate $\delta x_{k}^{+}$ is given as

(45) $$\delta x_{k}^{+}=\delta x_{k}^{-}+K_{k}\left(\delta z_{k}-H_{k}\delta x_{k}^{-}\right)$$

The expression $\left(\delta z_{k}-H_{k}\delta x_{k}^{-}\right)$ is referred to as innovation. It is the difference between the error of observation and its expected error, represented by $s_{k}$ as

(46) $$s_{k}=\delta z_{k}-H_{k}\delta x_{k}^{-}$$

The posteriori error-state covariance $P_{k}^{+}$ is given as

(47) $$P_{k}^{+}=\left(I-K_{k}H_{k}\right)P_{k}^{-}$$

The complete corrected navigation state ${\hat{x}}_{k}^{+}$ can be written as the sum of the error estimate from Equation (45) and prior full state estimate ${\hat{x}}_{k}^{-}$ as

(48) $${\hat{x}}_{k}^{+}={\hat{x}}_{k}^{-}+\delta x_{k}^{+}$$

The major assumption for obtaining proper results from ESKF is that the time interval should be short for error calculation and nonlinearity should not be dominant in the calculation of the innovation term. To compensate the effect of nonlinearity in the innovation Equation (46), we propose to modify it by incorporating an RBF neural network. The modified innovation term ${\tilde{s}}_{k}$ is given as

(49) $${\tilde{s}}_{k}=s_{k}-W_{k}y_{k}$$

where the term $W_{k}y_{k}$ is the output of the RBF neural network. The term $y_{k}$ is the output of the hidden layer of the RBF neural network and $W_{k}$ is the weight matrix that provides the link between output and hidden layers of RBF neural network. These weights can be designed by minimizing the the mean square error (MSE) cost function J, defined by

(50) $$J={∥s_{k}-W_{k}y_{k}∥}^{2}$$

To improve the estimation of multi-sensor fusion under nonlinear conditions, the Gaussian RBF function utilize an a priori error-state estimate, which is given as

(51) $$y_{k}\left(i\right)=\exp\left(-\frac{{∥\delta x_{k}^{-}-c_{ik}∥}^{2}}{2\sigma_{i}^{2}}\right),\;i=1,2,\ldots ,N_{c}$$

where $c_{ik}$ is the neuron center and $\sigma_{i}$ is the width of the Gaussian RBF function. The weights of the neurons in matrix form $w_{k}$ are represented as

$$W_{k}=\left(\begin{array}{cccc} w_{1k}\left(1\right) & w_{1k}\left(2\right) & \cdots & w_{1k}\left(N_{c}\right)\\ w_{2k}\left(1\right) & w_{2k}\left(2\right) & \cdots & w_{2k}\left(N_{c}\right)\\ \vdots & \vdots & \ddots & \vdots \\ w_{Mk}\left(1\right) & w_{Mk}\left(2\right) & \cdots & w_{Mk}\left(N_{c}\right) \end{array}\right)$$

where *M* donates the size of the state vector and $N_{c}$ represents the number of neuron centers. The size of $W_{k}$ is M×Nc. The size of $W_{k}y_{k}$ is M×1, and the size of $y_{k}$ is $N_{c}\times 1$.

### 5.2. Derivation of Weight Update of RBF-ESKF

For the weight matrix $W_{k}$, we only consider weight associated with the *m*th-specific output. Thus, the weight vector of the *m*th output of RBF can be denoted by $w_{mk}$. Therefore, the weight update rule of the specific *m*th output is given as

(52) $$w_{mk+1}=w_{mk}-\eta_{w}\frac{1}{2}\frac{\partial J}{\partial w_{mk}}$$

where $\eta_{w}$ is the learning rate, and its value is selected by experimentation. Too small a value of $\eta_{w}$ can cause unsuitability, and too large a value can make the response sluggish. A gradient descent method, namely the steepest decent, is used to minimize the cost function J relative to the RBF neural network weight. By using Equation (50), the gradient of cost function can be written as

(53) $$\frac{\partial J}{\partial w_{mk}}=\frac{\partial {∥s_{k}-W_{k}y_{k}∥}^{2}}{\partial w_{mk}}$$

The result of the derivative is given as

(54) $$\frac{\partial J}{\partial w_{mk}}=-2\left(\delta z_{k}\left(m\right)-H_{k}\left(m\right)\delta x_{k}^{-}\left(m\right)-w_{mk}y_{k}\right)y_{k}^{T}$$

Thus, putting Equation (54) in Equation (52), we get the complete weight update equation:

(55) $$w_{mk+1}=w_{mk}+\eta_{w}\left(\delta z_{k}\left(m\right)-H_{k}\left(m\right)\delta x_{k}^{-}\left(m\right)-w_{mk}y_{k}\right)y_{k}^{T}$$

### 5.3. Derivation of Center Update of RBF-ESKF

The center update rule for the *j*th element of the *m*th neuron center is given as

(56) $$c_{ik+1}\left(j\right)=c_{ik}\left(j\right)-\eta_{c}\frac{1}{2}\frac{\partial J}{\partial c_{ik}\left(j\right)}$$

where $\eta_{c}$ is the learning rate of center update. It is determined experimentally. By applying the steepest descent method, we minimized cost function J with respect to weight. The result of derivative is shown in the following equation:

(57) $$\begin{array}{c} \frac{\partial J}{\partial c_{ik}\left(j\right)}=-2\sum_{i=1}^{M}\left(s_{k}\left(m\right)+w_{mk}y_{k}\right)\cdot \left(\frac{w_{mk}\left(i\right)y_{k}\left(i\right)\left(\delta x_{k}^{-}\left(j\right)-c_{ik}\left(j\right)\right)}{\sigma_{i}^{2}}\right) \end{array}$$

Plugging Equation (57) into Equation (56), the new center update equation becomes

(58) $$\begin{array}{c} c_{ik+1}\left(j\right)=c_{ik}\left(j\right)+\eta_{c}\sum_{i=1}^{M}\left(s_{k}\left(m\right)+w_{mk}y_{k}\right)\cdot \left(\frac{w_{mk}\left(i\right)y_{k}\left(i\right)\left(\delta x_{k}^{-}\left(j\right)-c_{ik}\left(j\right)\right)}{\sigma_{i}^{2}}\right) \end{array}$$

It is evident from the weight update Equation (55) and center update Equation (58) that our algorithm uses system information to train the RBF neural network to overcome the drawbacks of ESKF.

**Figure 3:**
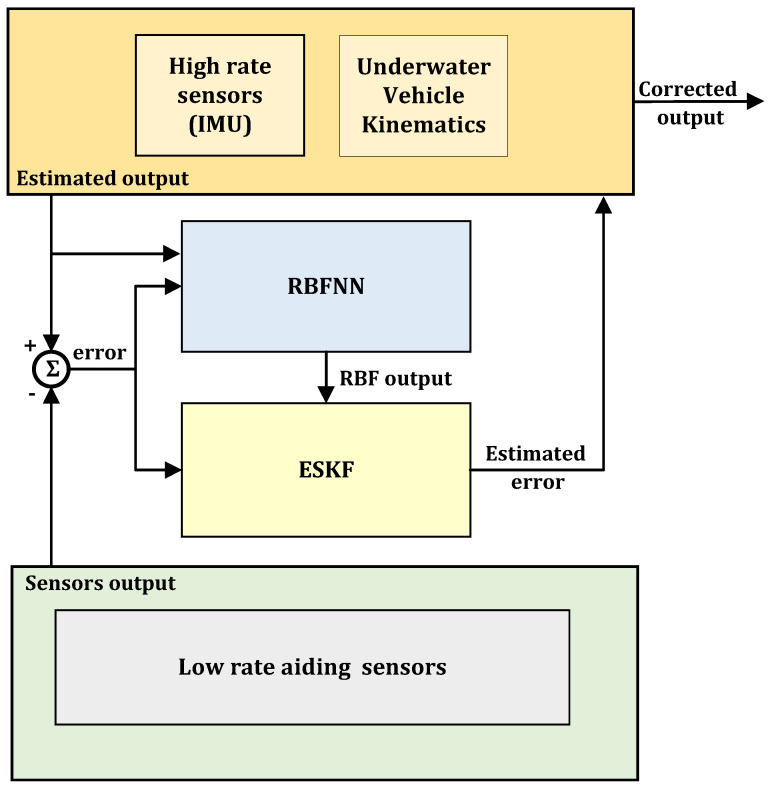
Complementary form representation of RBF-ESKF.

The complementary form block diagram representation of the RBF-ESKF fusion algorithm is shown in Figure 3. The vehicle kinematics combined with IMU high-rate sensors of IMU provide the estimated output. This estimated output is then subtracted from low-rate sensors and fed into RBF-ESKF. RBF and ESKF work together to find the best error estimate, which is then added into the underwater vehicle kinematics in feed-forward fashion to obtain the total state.

Algorithm 1 shows iterative steps of the proposed method. The term ${\tilde{s}}_{k}$ is RBF modified innovation term used by RBF-ESKF to improve accuracy of filter in highly non-linear conditions.
**Algorithm 1** RBF-ESKF multi-sensor fusion for underwater navigation   **Initialization:**1:Initialize ESKF variables $x_{0}^{-},P_{0}^{-},Q_{0},R_{0}$2:Initialize RBF variables $w_{0}^{,}c_{0},\sigma_{0},\eta_{w},\eta_{c}$   **Kalman gain update:**3:calculate Kalman gain
$K_{k}=P_{k}^{-}H_{k}^{⊤}{\left(H_{k}P_{k}^{-}H_{k}^{⊤}+R_{k}\right)}^{-1}$   **RBF Gaussian function update:**4:Learning non-linearity of error state vector
$y_{k}\left(i\right)=\exp\left(-\frac{{∥\delta x_{k}^{-}-c_{ik}∥}^{2}}{2\sigma_{i}^{2}}\right),\;i=1,2,\ldots ,N_{c}$   **Innovation update:**5:Non-linearity influence is minimized by using output of RBF neural network in innovation term
${\tilde{s}}_{k}=\delta z_{k}-\left(H_{k}\delta x_{k}^{-}+W_{k}y_{k}\right)$   **Measurement update:**6:Estimate error state by using innovation term and Kalman gain
$\delta x_{k}^{+}=\delta x_{k}^{-}+K_{k}{\tilde{s}}_{k}$7:Error state covariance update.
$P_{k}^{+}=\left(I-K_{k}H_{k}\right)P_{k}^{-}$   **Full State correction**8:Full state is corrected by error adding error estimate.
$x=\hat{x}+\delta x_{k}^{+}$   **RBF Neural Network Weight and Center update:**9:RBF weight update
$w_{mk+1}=w_{mk}+\eta_{w}\left(\delta z_{k}\left(m\right)-H_{k}\left(m\right)\delta x_{k}^{-}\left(m\right)-w_{mk}y_{k}\right)y_{k}^{T}$10:RBF center update
$$\begin{array}{c} c_{mk+1}\left(j\right)=c_{ik}\left(j\right)+\eta_{c}\left(\frac{w_{ik}\left(j\right)y_{k}\left(j\right)c_{ik}\left(j\right)}{\sigma_{i}^{2}}\right).\sum_{i=1}^{M}\left(\delta z_{k}\left(i\right)-\left(H_{k}\left(i\right)\delta x_{k}^{-}\left(i\right)+w_{ik}y_{k}\right)\right) \end{array}$$  **Time propagation:**11:Time propagation of error state and covariance
$$\delta x_{k+1}^{-}=\Phi_{k}\delta x_{k}^{+}$$
$P_{k+1}^{-}=\Phi_{k}p_{k}^{+}\Phi_{k}^{⊤}+Q_{k}$12:Next iteration (posterior becomes prior)

### 5.4. Complexity of RBF-ESKF

The proposed algorithm uses RBFNN to enhance the performance of underwater vehicle localization with a slight increased in time and space complexity compared to ESKF due to matrix multiplications. Compared to BPNN and deep learning neural networks, RBFNN has less complexity because of its simple three-layer structure because the time and memory space complexity of the neural networks is directly related to the structure and number of layers. However, faster matrix multiplication algorithms such as the Strassen algorithm [65] can be used to decrease execution time. Table 2 shows the structure of the RBFNN used in this work.

## 6. Results and Discussion

In order to compare performances, the proposed algorithm and ESKF were simulated in three different realistic scenarios. As the ESKF structure was modified by RBF in our fusion algorithm, low-level functions were written for simulation. The noise specifications of the sensors used in this work are comparable to their datasheets. The main purpose of the simulation was to compare the maximum error (max) and root means square error (RMSE) of position, velocity, and attitude. Practical failure mode tests cases were developed and simulated with DVL and acoustic positioning loss of measurements for a short duration. The simulation results were compared with conventional ESKF for performance evaluation. Furthermore, for simulation, the assumption was made that underwater vehicles can move in any direction and with any roll, pitch, and yaw angle. A reference trajectory of the vehicle was generated by angular velocities and acceleration.

To consider the effects of random variations in the accuracy of the fusion algorithms, a Monte Carlo simulation was used. The test consisted of 100 runs. Two filters processed the same data during the test to ensure a fair contrast. For all three cases, the same RBF neural network structure was used, as listed in Table 2. For the simulation, the RBF weights, centers, and sigma were initialized randomly.

### 6.1. Test Case 1: Normal Working Condition

In the first case, the vehicle was considered to be working in a normal operating mode without any on-board and off-board sensor failure. To simulate a real situation, the noise and drift characteristics of the sensors used for simulation were almost the same as listed in the manufacturer’s documentation stated under Section 3. Both ESKF and RBF-ESKF were tested on the same operating conditions. The performances of ESKF and RBF-ESKF are compared side by side in Table 3.

From the results of the north position prediction displayed in Table 3, it can be observed that ESKF has an almost three times higher maximum error than that of the RBF-ESKF. Furthermore, for the east position, the ESKF maximum error was twice as high as the RBF-ESKF. In the case of maximum error in the prediction of depth, the performance of the two filters are approximately identical. The RBF-ESKF RMSE for position estimation was even better, almost twice as high. Significant improvement was seen in the north, where it were three times higher than the one achieved by the ESKF. Overall, the RMSE sum for the RBF-ESKF position estimate was approximately two times better than that of the ESKF.

The estimation of velocity showed considerable improvement compared to the ESKF. The overall northern velocity error was almost double than that of the ESKF compared to the RBF-ESKF. Small but noticeable improvements were seen in the maximum error for east and depth velocities. The RMSE of the RBF-ESKF was roughly two times better than the ESKF for the north, east, and down velocities. Overall, with our proposed fusion algorithm, the sum of all RMSE states was almost three times better than ESKF.

Overall, in terms of attitude, relative to ESKF, the sum of all state RMSEs improved significantly by about double as much.

**Figure 4:**
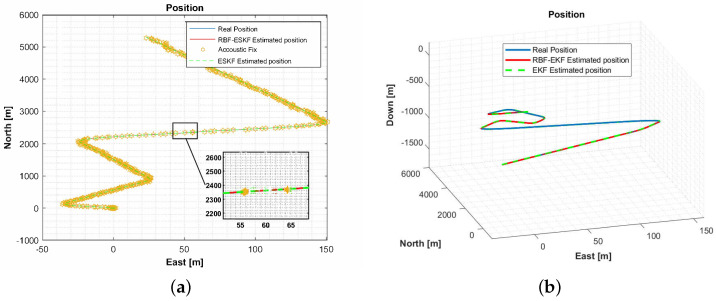
(**a**) A comparison of the 2D trajectory of an underwater vehicle in the east and north directions and (**b**) a comparison of the 3D trajectory of an underwater vehicle.

Figure 4 shows the 2D and 3D trajectories. It can be seen that the trajectory is not linear. The position, velocity, and attitude errors are estimated on this trajectory. The star symbol in the 2D trajectory shows an acoustic fix from the external source. The performances of these ESKF and ESKF-RBF were evaluated by how close the estimated path was to the actual value.

**Figure 5:**
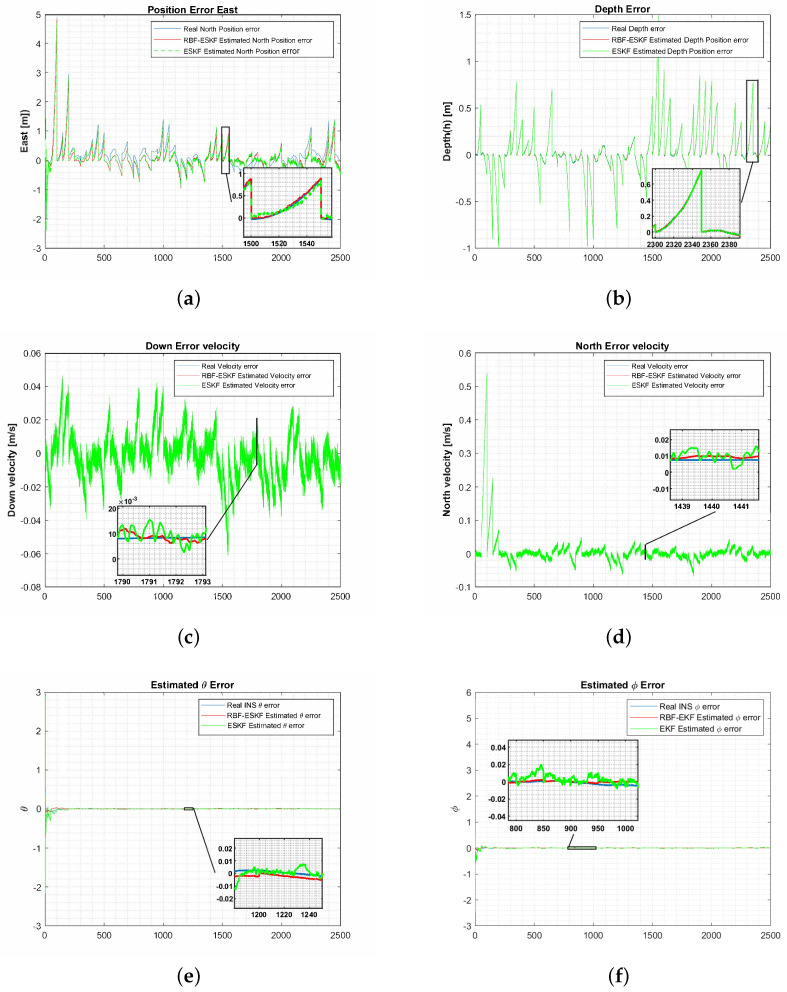
Simulation results of ESKF and RBF-ESKF for time 0–2500 s (x-axis) in case 1. (**a**,**b**) The RBF-ESKF estimation for the east positions and depth error is close to the actual error value. (**c**,**d**) Error velocity for down and north. (**e**,**f**) Euler angle errors for roll and pitch. It is evident from (**c**–**f**) that ESKF has an oscillatory response that contributes to compromised accuracy compared to RBF-ESKF.

Figure 5 shows comparison of ESKF and RBF-ESKF estimated the position, velocity and attitude.

### 6.2. Test Case 2: Acoustic Fix Not Available

In this case, the robustness of the multi-sensor fusion algorithm was tested by simulating the loss of the underwater acoustic fix for a short period. The unavailability of the position information from acoustic fix mostly influenced the position estimate of both filters, predominantly ESKF. In this case, the position estimate was only available from the integration of the DVL velocity, which compensated the acoustic fix loss effect and reduced the drift error. The detailed comparison is shown in Table 4.

It can be noted from Table 4 that the maximum error of RBF-ESKF was almost five times better for the north and east directions as compared to ESKF. The down position estimate was also two times better than that of ESKF. The overall RMSE in all three directions is considerably improved by almost one and a half times that of ESKF. Overall, compared to normal working conditions, the position accuracy had a detrimental effect, but RBF-ESKF has proven to be more robust.

In contrast to standard operating conditions, the velocity estimation was marginally influenced by acoustic fix unavailability. The maximum ESKF error was worse than that of RBF-ESKF for velocity in all directions. For RBF-ESKF, the RMSE of velocity was significantly better by almost two times that for ESKF. Overall, with our proposed method of fusion, the sum of all RMSE velocity states was almost two times better than ESKF.

For RBF-ESKF, the RMSE of the roll, pitch, and yaw angles were substantially better by about one and a half times that of ESKF. The maximum roll, pitch, and yaw angle errors were two times better for RBF-ESKF. Overall, the sum of all state for RBF-ESKF was better than that for ESKF.

**Figure 6:**
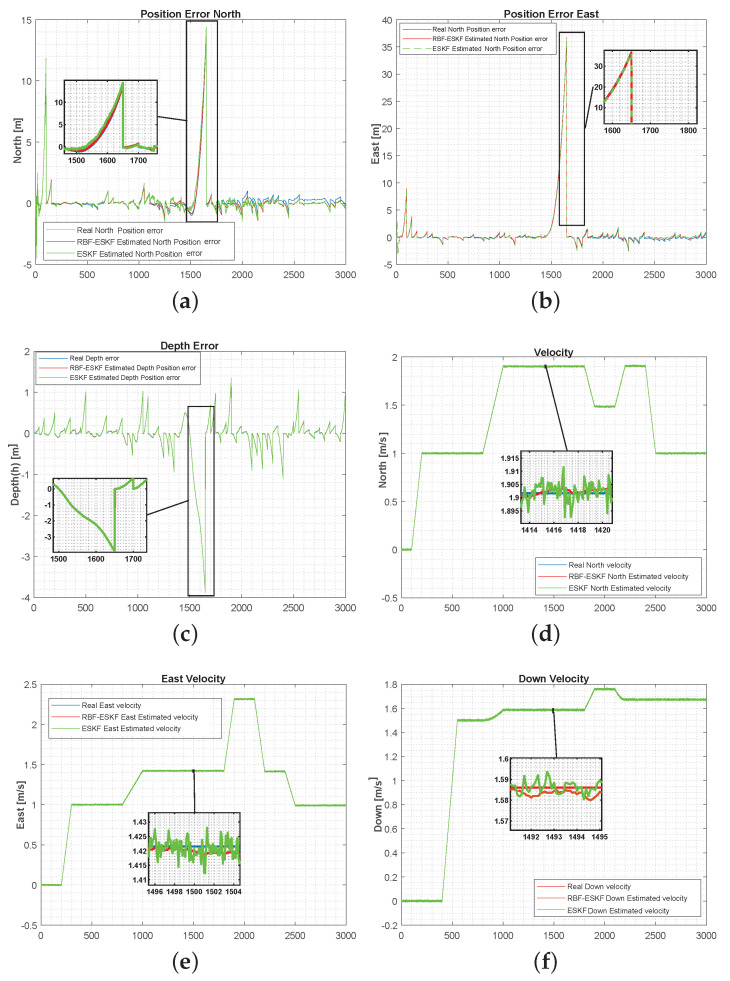
Simulation results of ESKF and RBF-ESKF for time 0–3000 s (x-axis) in case 2. (**a**–**c**) The position error significantly grows with a loss of acoustic fix. The proposed algorithm estimation is close to the actual error. (**d**–**f**) RBF-EKF has a minimal effect of acoustic fix loss on velocity estimation, and the response is much smoother than that of ESKF.

Figure 6 below illustrates a comparison of the position and velocity errors when acoustic fix was not available for a short period from 1500 to 1700 s. The performances of ESKF and ESKF-RBF were evaluated on similar trajectory. Moreover, the RBF-EKF is consistent in the estimation and is close to the actual value.

### 6.3. Test Case 3: DVL Unavailable

In this case, the robustness of both filters was tested when DVL was not available for a short duration. The velocity estimate had a larger influence than the position estimate because, when DVL was not available, it was calculated by taking the derivative of the position measurement, which suffers from noise amplification from the differentiation process. Moreover, acoustic fix measurement bias was negatively influenced by DVL measurements that contribute to increasing the position error. Table 5 compares the performance robustness multi-sensor fusion algorithm with DVL failure.

It can be observed from the above data that position estimate in this case was better than case 2 but slightly less accurate than normal working conditions. However, in the north direction, ESKF maximum error was almost two times worse and almost one and a half times worse for east and depth. Furthermore, the RBF-ESKF RMSE for the position was notably better than that for ESKF by around one and a half times in all directions. Thus, the position of estimation for the overall mission was improved in all directions by the RBF-ESKF algorithm.

The velocity estimation without DVL was less accurate compared to case 1 and case 2. However, the overall results of our method are much better than those of ESKF, which were almost doubly improved with respect to the maximal error and RMSE. In addition, the sum of all estimated velocity states from RBF-ESKF in all directions was approximately two times better than that of ESKF. Hence, the RBF-ESKF velocity estimation was more robust than that of ESKF. The RBF-ESKF attitude estimation of the roll, pitch, and yaw angles had a lower maximum estimation error. Moreover, the RMSE for RBF-ESKF showed considerable improvement. Overall, the sum of all estimated attitude states was one and half times better than that of ESKF.

**Figure 7:**
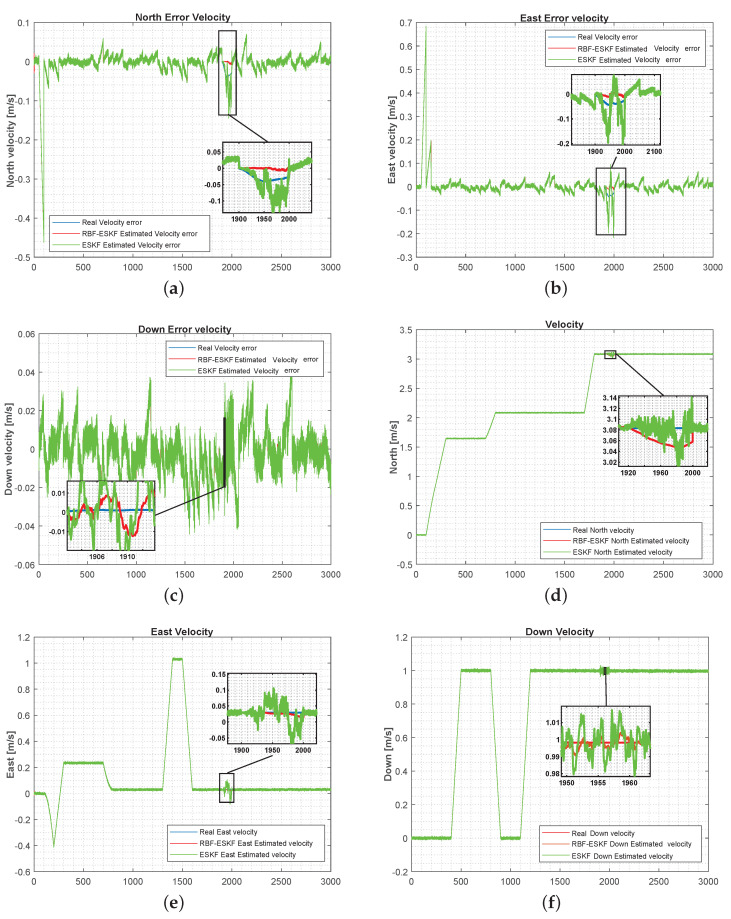
Simulation results of ESKF and RBF-ESKF for time 0–3000 s (x-axis) in case 3. As the error increases, nonlinearity in the error also increases. (**a**–**c**) The velocity error for the north, east, and downward directions increase significantly with DVL loss. The proposed velocity error estimate of the algorithm is similar to real error. (**d**–**f**) RBF-ESKF has a smoother response and faster convergence of the full state velocity estimation.

Figure 7 below shows comparison velocity errors and velocity when the DVL measurement was not available for a short duration from 1900 to 2000 s. The performances of ESKF and ESKF-RBF were evaluated on a similar trajectory as test case 1.

The time complexity of ESKF and ESKF-RBF was tested by running the algorithms on an Intel I7 CPU with 4 GB RAM without any GPU. The testing software used was Matlab 2020b on Windows 7 platform. The timing comparison of both methods is given in Table 6.

On a high-speed microcontroller or field-programmable gate array FPGA, the execution time difference will be further reduced. Furthermore, the speed of surveying the type of underwater vehicle is in the range of 2 km/h to 10 km/h; this difference has no significant effect on navigation and localization.

The work tried to fill the gap by proposing a noval ESKF and RBF-augmented fusion solution, which was assessed in three different underwater cases. The ESKF performance strongly relies on the knowledge of the system models and noise properties, which was degraded by nonlinearity. The errors in the RBF-ESKF are smaller than the errors in the ESKF because of the recurcive learning of RBF. Moreover, the fusion algorithm based on RBF-ESKF with help from the aiding sensors was able to correct the drift problems in the INS with better accuracy.

Nevertheless, the proposed algorithm is not limited to underwater; it can also be used in other applications [66,67] such as improving aircraft navigation and tracking by using aerial sensors. Moreover, autonomous ground vehicles are another area where this method can be employed. Furthermore, by improving the Kalman filer response, our methodology can also enhance accuracy satellite attitude estimation [68].

## 7. Conclusions

This paper discusses the performance of the proposed algorithm RBF-ESKF for underwater vehicle localization. The primary aim of this work is to take advantage of the RBF neural network to improve the estimation performance of the conventional ESKF for the position, velocity, and attitude of an underwater vehicle. It contrasts outcomes with ESKF in three separate simulations, showing that RBF-ESKF performs better in estimating position, velocity, and attitude. Why the standard ESKF has an inferior performance is because it is designed by employing first-order Taylor series approximation in the error covariance matrix estimation, which results in a decrease in estimation accuracy under high nonlinearity. Thus, important information about the dynamic of underwater is lost because of this realization. However, RBF-ESKF efficiently handles nonlinearity due to its inherent capability for nonlinear function approximation and learning ability.

The research also compared robustness in cases when there is no available position information from the acoustic fix. Here, relative to ESKF, RBF-ESKF demonstrated better accuracy. When acoustic fix becomes available, RBF-ESKF converges quickly. In addition, when DVL fails due to short durations, RBF-ESKF also demonstrates less estimation error.

This work is part of an ongoing work on underwater vehicle localization. The novel proposed algorithm is intended for use as a state estimation for underwater seabed mapping application.

In the future, we would like to test and analyze our algorithm with a different set of sensors with different data rates in underwater environments. For instance, an enhanced version of the multi-sensor fusion algorithm could be designed by using stereo vision and underwater wireless sensor nodes to improve localization.

## Figures and Tables

**Table 1:** Mathematical notations for underwater vehicle kinematics.

Notation	Description
$v^{b}$ [u,v,w]	Linear velocity in the body frame (surge, sway, and heave)
$\Theta_{nb}\left[\phi ,\theta ,\psi \right]$	Attitude in Euler angles from the body to NED frame
$q_{b}^{n}\left[\eta ,\epsilon \right]$	Attitude in quaternion from the body to NED frame
$\omega^{b}$ [p,q,r]	Angular velocity in the body frame (roll, pitch, and yaw)
$p^{n}$ [n,e,d]	Position in the NED frame (north, east, and down)
$v^{n}$ $\left[v_{N},v_{E},v_{D}\right]$	Linear velocity in the NED frame (north, east, and down)
$\omega^{n}$ $\left[\omega_{N},\omega_{E},\omega_{D}\right]$	Angular velocity in the NED frame
$p^{e}$ [x,y,z]	Position in the ECEF frame
$v^{e}$ $\left[v_{ex},v_{ey},v_{ez}\right]$	Linear velocity in the ECEF frame
$p^{e}\left[\phi_{lat},\lambda_{long},h_{d}\right]$	Position in the ECEF geoid (latitude, longitude, and depth)
$R_{b}^{e}$	Rotation matrix from the body to ECEF frame
Ω	Skew symmetric matrix of the angular velocity
$\Omega^{e}$	Skew symmetric matrix in the ECEF frame
$\Omega^{b}$	Skew symmetric matrix in the body frame
$g^{e}$	Earth gravity vector in the ECEF frame
$g^{n}$	Earth gravity vector in the NED frame
$R_{M}$	Radius of curvature of the prime vertical of Earth
$R_{N}$	Radius of curvature of the meridian of Earth
*a*	Semi-major axis of the ellipsoidal Earth model
*e*	Eccentricity of the ellipse approximation of Earth

**Table 2:** RBF neural network structure with a Gaussian activation function.

RBFNN	Numbers
Input layer neurons	20
Hidden layer neurons	50
output layer neurons	20
Learning rate of weights	0.001
Learning rate of centers	0.001

**Table 3:** ESKF and RBF-ESKF results with all sensors working in normal condition by running 100 Monte Carlo simulations.

	ESKF	RBF-ESKF
North Position Max error	1.1509	0.32578
East Position Max error	0.8218	0.45685
Down Position Max error	0.0081786	0.0071222
North Position RMSE	0.446124	0.1464
East Position RMSE	0.3122	0.1844
Down Position RMSE	0.0040719	0.002709
Sum Position RMSE	0.76239	0.333509
North Velocity Max error	0.038185	0.01844
East Velocity Max error	0.0045943	0.0037403
Down Velocity Max error	0.0032507	0.0022476
North Velocity RMSE	0.039455	0.011407
East Velocity RMSE	0.033652	0.012919
Down Velocity RMSE	0.0035526	0.020227
Sum Velocity RMSE	0.0766596	0.0263487
Roll Max error	0.13268	0.060874
Pitch Max error	0.1809	0.16171
Yaw Max error	0.46624	0.36001
Roll RMSE	0.00039554	0.00019122
Pitch RMSE	0.00055597	0.00036354
Yaw RMSE	0.00049874	0.00035049
Sum Attitude RMSE	0.00145025	0.00122578

**Table 4:** Performance Comparison of ESKF and RBF-ESKF with loss of acoustic fix for a short period by running 100 Monte Carlo simulations.

	ESKF	RBF-ESKF
North Position Max error	5.2115	1.3849
East Position Max error	1.4275	0.4663
Down Position Max error	0.033167	0.016192
North Position RMSE	0.94036	0.50629
East Position RMSE	0.66511	0.40109
Down Position RMSE	0.005613	0.0039411
Sum Position RMSE	1.611083	0.9113211
North Velocity Max error	0.040376	0.029489
East Velocity Max error	0.0036513	0.0030517
Down Velocity Max error	0.0061216	0.0036592
North Velocity RMSE	0.0439324	0.21818
East Velocity RMSE	0.065722	0.035722
Down Velocity RMSE	0.0065451	0.0039131
Sum Velocity RMSE	0.1161995	0.0614531
Roll Max error	0.2567	0.1231
Pitch Max error	0.41582	0.19178
Yaw Max error	0.5494	0.46001
Roll RMSE	0.0006135	0.00020485
Pitch RMSE	0.00052358	0.00011917
Yaw RMSE	0.00051363	0.0003413
Sum Attitude RMSE	0.00169071	0.00117532

**Table 5:** Performance comparison of ESKF and RBF-ESKF with the Doppler velocity log (DVL) measurement unavailable for a short duration by running 100 Monte Carlo simulations.

	ESKF	RBF-ESKF
North Position Max error	1.9455	0.90707
East Position Max error	0.91649	0.63511
Down Position Max error	0.0084106	0.0072865
North Position RMS error	0.669324	0.41818
East Position RMS error	0.53722	0.30722
Down Position RMS error	0.0049455	0.0035131
Sum Position RMS error	1.2114895	0.7289131
North Velocity Max error	0.105751	0.051603
East Velocity Max error	0.099018	0.061305
Down Velocity Max error	0.0178454	0.00897
North Velocity RMS error	0.191296	0.09809
East Velocity RMS error	0.15337	0.079982
Down Velocity RMS error	0.0092455	0.005387
Sum Velocity RMS error	0.3539115	0.183459
Roll Max error	0.29935	0.171758
Pitch Max error	0.51906	0.210442
Yaw Max error	0.66041	0.42246
Roll RMSE	0.00066481	0.000450075
Pitch RMSE	0.00060017	0.00042717
Yaw RMSE	0.00062205	0.000422102
Sum Attitude RMSE	0.00188703	0.001299347

**Table 6:** Execution time (seconds).

ESKF	ESKF-RBF
0.0028	0.0039

## Data Availability

Not applicable.
